# Ten years of battle with multiple recurrences of pediatric skull base chondrosarcoma: A case report

**DOI:** 10.1002/ccr3.4904

**Published:** 2021-10-04

**Authors:** Mona Ariamanesh, Rana Tafrishi, Mansoureh Dehghani, Mehdi Bakhshai, Seyed Alireza Javadinia, Seyyed Morteza Hosseini, Mohammad Nematshahi, James S. Welsh, Pejman Porouhan

**Affiliations:** ^1^ Neyshabur University of Medical Sciences Neyshabur Iran; ^2^ Department of Pediatrics Faculty of Medicine Mashhad University of Medical Sciences Mashhad Iran; ^3^ Department of Otolaryngology Qaem Hospital Mashhad University of Medical Sciences Mashhad Iran; ^4^ Clinical Research Development Unit Hospital Research Development Committee Sabzevar University of Medical Sciences Sabzevar Iran; ^5^ Razavi Cancer Research Center, Razavi Hospital Mashhad Iran; ^6^ Department of Anesthesiology and Critical Care Sabzevar University of Medical Sciences Sabzevar Iran; ^7^ Edward Hines Jr VA Hospital and Loyola University Chicago Stritch School of Medicine Chicago Illinois USA; ^8^ Department of Radiation Oncology Vasei Educational Hospital Sabzevar University of Medical Sciences Sabzevar Iran

**Keywords:** chondrosarcoma, proptosis, skull base

## Abstract

In children and adolescents presenting with skull base sarcoma, treatment strategies will face challenging decisions due to the unique chemoresistant pathologies, limitations imposed by the not‐yet fully mature anatomical structures, and the small surgical site.

## Graphical abstract

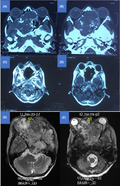

## 1 INTRODUCTION

In this report, we present the case of a 17‐year‐old male with a history of multiply recurrent skull base sarcomas that began at the age of 7. Long‐term survival is achievable in these rare conditions even after multiple recurrences, but success demands a multidisciplinary team.

Head and neck sarcomas comprise less than 1% of all head and neck malignancies.^1^ Sarcomas with skull base involvement are associated with poor local control rates because of the complex cranial anatomy and difficulty in delivering intensive treatments.^2^

Sarcomas constitute a heterogeneous group of malignancies that vary extensively by anatomic location, histology, and biologic behavior. A multidisciplinary approach including surgery, radiotherapy, and, in chemosensitive histopathologies, chemotherapy is necessary. Chondrosarcoma is a neoplastic process associated with cartilage matrix production lacking osteoid, a characteristic of osteosarcoma. Chondrosarcoma may arise at any age but typically occurs in middle‐aged and older adults between the age of 20 and 60 years old, and it is more common in men. It can also develop from malignant transformation in benign cartilaginous lesions such as enchondroma or osteochondroma.^3^

Surgical excision is the main treatment option for chondrosarcoma. For low‐grade tumors, which constitute the majority of these tumors, surgical resection alone is sufficient to achieve a high rate of disease control. For low‐grade central tumors, intralesional excision or curettage can be combined with local adjuvant chemical treatment, cryotherapy, or radiation. These approaches often result in good local control.^4^, ^5^, ^6^

Skull base tumors in children and adolescents impose greater therapeutic challenges. Although many treatment options for such tumors in adults have been suggested, relatively little is reported with regards to therapies in the pediatric population. The unique pathological composition, the limitations imposed by the maturing skull and brain, and the small size of the patients, make it more challenging to select the best approach.^7^

## 2 CASE PRESENTATION

**FIGURE 1:**
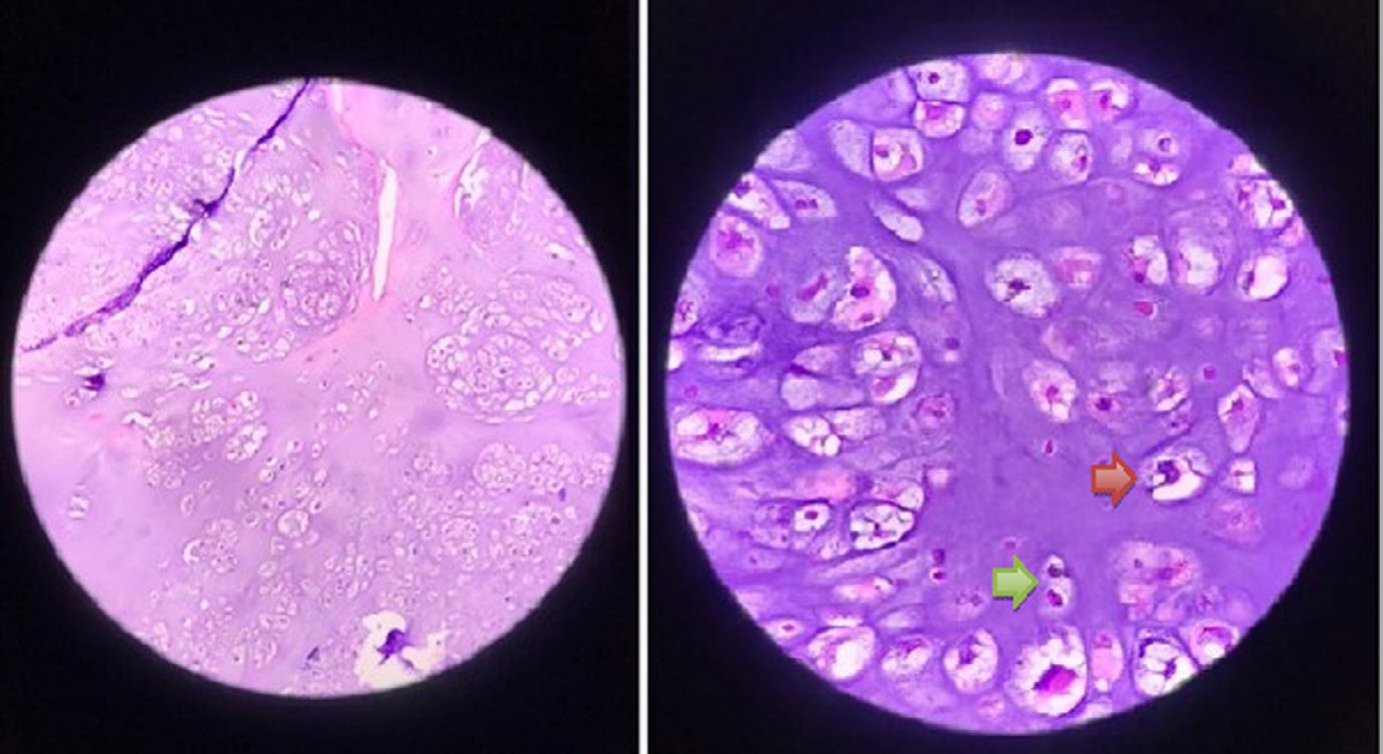
Microscopic tumor morphology in favor of a diagnosis of low‐grade chondrosarcoma. (green arrow: binucleation of chondrocytes, red arrow: large, highly atypical chondrocyte) (Left: Low Power Field, Right: High Power Field)

The patient first presented to the surgery clinic in 2010 at the age of 6 with a chief complaint of an exacerbation of left eye proptosis, which had been previously progressing slowly. Brain magnetic resonance imaging (MRI) revealed a tumoral lesion in the infratemporal skull base and maxillary sinus, with sphenoid spread and optic nerve compression. Endoscopic transnasal resection of tumor, left orbital decompression and infratemporal space evacuation was performed. The tumor grossly appeared chondroid and some degree of clivus bone destruction was identified intraoperatively. A pathologic examination demonstrated low‐grade chondrosarcoma, and the patient was discharged with follow‐up recommendation (Figure 1).

Follow‐up imaging (MRI) four months later, revealed recurrence in the right maxillary sinus and endoscopic surgery was planned. Ophthalmologic consultation before surgery demonstrated left eye visual acuity of 1/10 and right eye visual acuity of 10/10.

Resection of the tumoral tissue located in the floor, medial, and posterior walls of the maxillary sinus was accomplished with negative margins via endoscopic approach, but complete resection of the superior aspect was not feasible.

One year later, following another recurrence, MRI revealed tumor presence in the sinonasal area and skull base without involvement of the brain parenchyma. Endoscopic resection of maxillary sinus and skull base was done. The right optic nerve was involved by the tumor and a resection of the medial and inferior orbital wall was also required.

Over the next 2 years, two more recurrences occurred along with some erosion of the clivus bone, which resulted in the patient's blindness due to optic nerve atrophy. Both recurrences were treated with double surgical resections.

After 9 recurrences during a period of 10 years, another follow‐up imaging displayed abnormal heterogeneous foci on the left temporal lobe, left retro orbital area, and left parasellar region, with extension to nasal cavity. Contrast‐enhanced imaging demonstrated a 70 × 60 mm multiloculated mass. The case was discussed in the neurosurgery multidisciplinary tumor board and tumor debulking plus radiotherapy was recommended. The patient did not follow the last recommendation and returned to the clinic after 19 months, with massive tumor progression which was referred to a neurosurgeon and was announced unresectable due to fatal surgical risk. He was then referred for palliative radiotherapy which consisted of 20 fractions of 250 (centigray) cGy per fraction (total dose = 50 Gy).

Due to the patient's excessive suffering from ocular edema, deformity, and eye discharge, right ocular evisceration was performed after completion of radiotherapy. Similar problems in the left eye gradually regressed after treatment.

**FIGURE 2:**
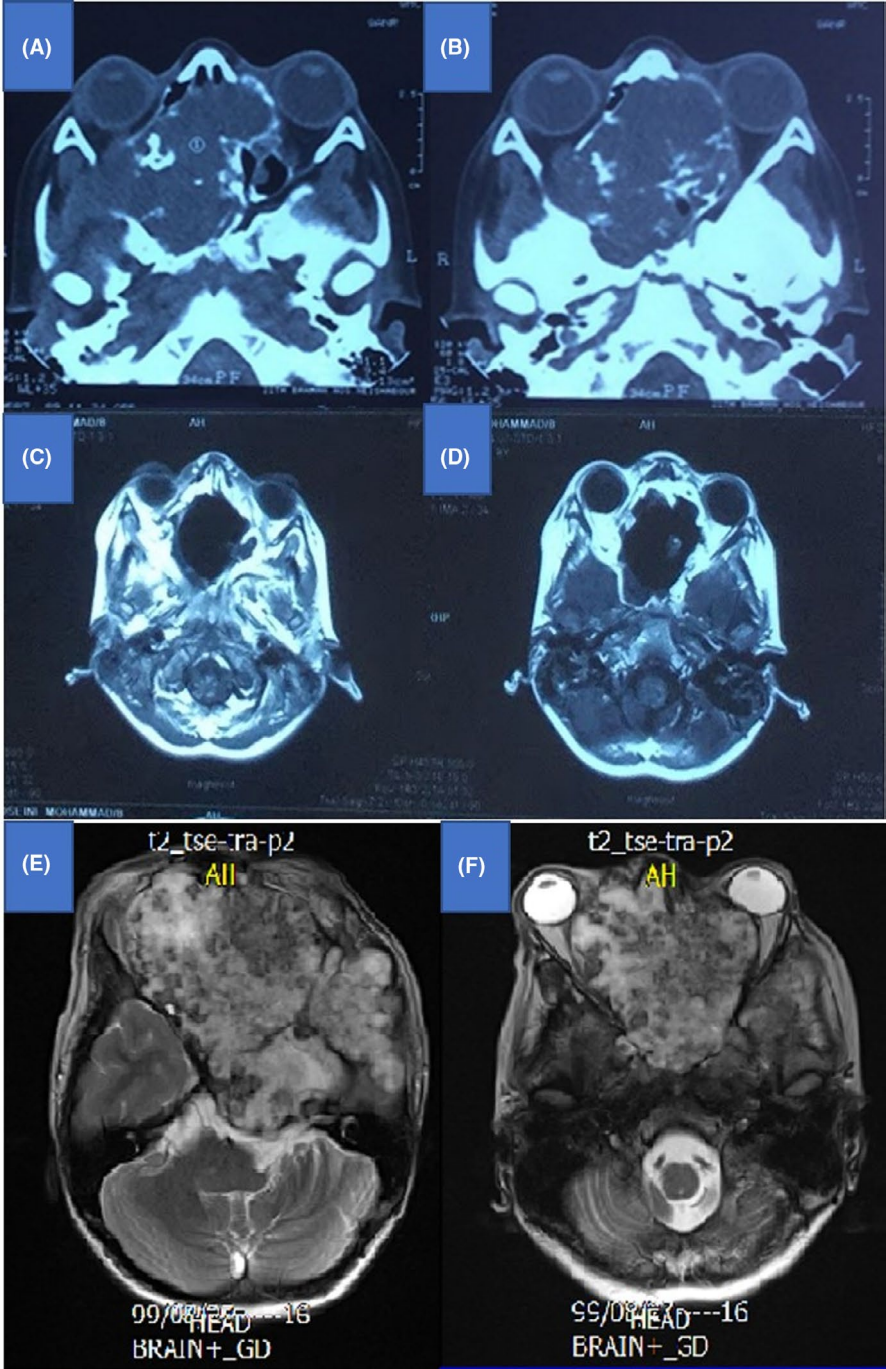
(A) Initial CT imaging indicating the diagnosis of skull base tumor. (B) Post‐surgical changes and complete excision demonstrated in the first post‐surgical CT imaging. (C) Final MRI imaging indicating a large heterogeneous tumoral mass which remained stable after radiation therapy

Currently, the patient is under follow‐up and the tumor, despite being bulky and locally advanced, has been in a stable status for 6 months (Figure 2). Both eyes are blind, but he has normal cognitive function, intelligence quotient (IQ), gait and sensory‐motor performance.

## 3 DISCUSSION

Malignant tumors of the skull base are rare and pathologically diverse neoplasms. These tumors may arise from the bony skull base or from the intracranial compartments, but more frequently, they arise from the subcranial tissues.^8^ These tumors commonly arise from and involve structures such as the infratemporal fossa, paranasal sinuses, and nasopharynx and are rarely completely resected by using traditional neurosurgical approaches. Thus, a multidisciplinary team comprising different approaches and modalities is ideal to address these tumors in a radical fashion.^9^

Although there are a limited number of studies on skull base sarcomas, it is reported that among patients with sarcoma, those with tumors located in the skull base region have the lowest overall survival (OS) rates.^10^ Studies have reported a 5‐year OS rate of 75% and a progression‐free survival rate at 5 years of 60%.^11^ Case series reported that approximately 50% of patients who fail will have done so within 2 or 3 years after the completion of treatment. Unlike sarcomas in other anatomic sites, in which distant metastases are the most common cause of death, the majority of deaths in patients with skull base tumors are related to local recurrence at the skull base that involves vital structures, such as the cavernous sinus, main arteries, and brain.^12^ In the present report, the patient has experienced frequent recurrences with an average interval of 1 year, ultimately resulting in optic nerve atrophy but with normal brain function despite the presence of an extensive remnant.

In an international collaborative study in 2007, Gil et al. evaluated 146 patients with anterior skull base sarcoma in terms of clinical manifestations and prognostic factors. Orbital involvement, involvement of the orbital wall, and intracranial extension were reported in 53%, 46%, and 28% of the patients, respectively. Tumor grade and adjuvant radiotherapy were not significant predictors of survival. Prior radiotherapy, intraorbital extension, positive margins, and postoperative complications were significant predictors of reduced disease‐specific survival on univariate analysis. The presence of positive or close margins was the only independent predictor of poor overall, recurrence‐free, and disease‐specific survival on multivariate analysis. The authors concluded that wide craniofacial resection with negative margins is an important predictor of better treatment outcome.^12^

In a recent study with similar end‐points, Kobayashi et al., retrospectively reviewed 22 sarcomas with skull base invasion, and reported that cases whose surgical margins were classified as “wide margin positive” had significantly worse 3‐year local recurrence rate than did patients with “margin negative” or “micro margin positive” status (25% vs. 83%, *p* = 0.014). They also concluded that resection margin status was an independent predictive factor for local recurrence after radical skull base surgery, and conservative surgery is only acceptable in chemosensitive subtypes.^2^

Mandonnet et al., retrospectively reviewed 42 patients younger than 19 who underwent surgery for a skull base tumor between 1992 and 2002, and analyzed the disease spectrum and outcomes. The median age was 13.75 years, and twenty‐two patients harbored a malignant tumor. Gross total resection was achieved in 78% of cases and local recurrence rate was 47%. With the median follow‐up being 63 months, 14% of patients had a neurological deficit and mortality rate was 30%. Middle cranial base tumors resulted in poorer prognosis.^13^ It is worth noting here that our patient had an anterior skull base tumor and his age at first presentation was young. Some studies have indicated that outcomes in younger patients may be better, in part because of the benign histopathology that frequently affects the pediatric skull base, in addition to the plasticity of the maturing nervous system.^7^

Chondrosarcoma is a neoplastic process associated with cartilage matrix production and may arise in different locations of the body. Patients typically present with localized pain in the affected bone with or without involvement of the adjacent soft tissue in the form of a palpable mass or tissue swelling.^3^ In our patient, however, the clinical presentation consisted of tissue swelling and ocular proptosis in the absence of a complaint of significant pain. Histologic grade and tumor location are believed to be the most important factors defining the clinical presentation and treatment approaches.^3^ Although the main treatment is complete surgical excision and radiotherapy is mostly reserved for multiple recurrences or unresectable tumors, upfront adjuvant radiotherapy has also been proposed in some studies. In a retrospective analysis of patients with low‐grade skull base chondrosarcoma, patients were divided according to post‐surgical strategies: planned upfront radiotherapy or watchful waiting. With a median follow‐up of 105 months, the 10‐year disease‐specific survival was 95% and there was no difference between the two groups. The majority of the patients in the latter group who experienced recurrence obtained tumor control with repeat resection, salvage radiotherapy, or both.^14^

Type of treatment center and access to health care facilities have a significant impact on mortality and survival for skull base chondrosarcoma. In a review of the National Cancer Database of the United States published in 2021, data analysis demonstrated that higher education and treatment at a high‐volume academic facility were associated with decreased 90‐day mortality for patients with skull base chordoma and chondrosarcoma. The authors stated that academic approach, radiotherapy, escalated doses, and proton radiation facilities were more likely to be available at high‐volume centers.^15^ In the present case, although all the surgical approaches were accomplished in high‐volume academic centers and the patient was followed by a multidisciplinary team, lack of technologies such as proton beam radiation in the country led to the deferral of radiotherapy until extensive progression. However, according to present evidence, the perfect timing to implement radiation and the cost‐value balance in complications is not fully understood.^14^, ^16^ In confirmation of the above results about high‐volume academic centers, it is worth mentioning that a recent report by Memorial Sloan Kettering Cancer Center, 31 patients with skull base sarcomas treated with primary surgery were evaluated and a 5‐year disease‐specific survival of approximately 70% was stated.^17^

Despite the advances in imaging and diagnostic techniques, surgical techniques that incorporate oncologic principles, conformal radiation facilities, potential targeted systemic therapies, and early access to coordinated multidisciplinary team, extensive skull base chondrosarcoma, especially in cases of intracranial extension portends worse prognosis and lower survival rates.^18^, ^19^

## 4 CONCLUSION

In children and adolescents presenting with skull base sarcoma, treatment decisions are challenging due to the unique and frequently chemoresistant pathologies, treatment limitations imposed by the immature anatomical structures, and the small size of the patients. Chondrosarcoma may arise at any age but is rare in children and adolescents. It is important to manage these rare diseases through a multidisciplinary team and with a perspective toward the future recurrences and available options. Long term survival is achievable even after multiple recurrences and surgeries, when approached via a multidisciplinary team.

## CONFLICTS OF INTEREST

Authors declare that they have no conflicts of interest.

## AUTHOR CONTRIBUTIONS

M.A., M.B., and S.A.J contributed in conception, design and drafting of the manuscript. P.P, M.D., and S.M.H. contributed in data collection. S.M.H., M.N., and J.S.W contributed in drafting of the manuscript. S.A.J. supervised the study. All authors approved the final version for submission.

## ETHICAL APPROVAL

The study was approved by Sabzevar University of Medical Sciences. The study conforms to recognized standards is of Declaration of Helsinki. An informed written consent form was obtained from patient and his guardian and he signed informed consent regarding publishing his data and photographs anonymous.

## CONSENT

We would like to thank the patient and his guardian for his consent for publication of his data.

## Data Availability

The data sets used and/or analyzed during the current study are available from the corresponding authors per request.
